# Nitrogen-Doped Superporous Activated Carbons as Electrocatalysts for the Oxygen Reduction Reaction

**DOI:** 10.3390/ma12081346

**Published:** 2019-04-25

**Authors:** María José Mostazo-López, David Salinas-Torres, Ramiro Ruiz-Rosas, Emilia Morallón, Diego Cazorla-Amorós

**Affiliations:** 1 Department of Inorganic Chemistry and Materials Institute, University of Alicante, 03080 Alicante, Spain; mj.mostazo@ua.es (M.J.M.-L.); ramiro@uma.es (R.R.-R.); 2 Department of Physical Chemistry and Materials Institute, University of Alicante, 03080 Alicante, Spain; david.salinas@ua.es (D.S.-T.); morallon@ua.es (E.M.)

**Keywords:** porous carbons, polyaniline, nitrogen functionalization, electrocatalysis, oxygen reduction reaction

## Abstract

Nitrogen-containing superporous activated carbons were prepared by chemical polymerization of aniline and nitrogen functionalization by organic routes. The resulting N-doped carbon materials were carbonized at high temperatures (600–800 °C) in inert atmosphere. X-ray Photoelectron Spectroscopy (XPS) revealed that nitrogen amount ranges from 1 to 4 at.% and the nature of the nitrogen groups depends on the treatment temperature. All samples were assessed as electrocatalysts for the oxygen reduction reaction (ORR) in alkaline solution (0.1 M KOH) in order to understand the role of well-developed microporosity as well as the different nitrogen functionalities on the electrocatalytic performance in ORR. It was observed that nitrogen groups generated at high temperatures were highly selective towards the water formation. Among the investigated samples, polyaniline-derived activated carbon carbonized at 800 °C displayed the best performance (onset potential of 0.88 V versus RHE and an electron transfer number of 3.4), which was attributed to the highest concentration of N–C–O sites.

## 1. Introduction

Fuel cells have attracted considerable attention as energy conversion devices for stationary and mobile applications mainly due to their high efficiency and low emissions. However, the development of new catalysts for the oxygen reduction reaction (ORR) that takes place in the cathode is still a key challenge to expand this technology [1]. The most common and high-performing materials are based on platinum and other noble metals supported on carbon materials [2,3,4]. However, they experience several drawbacks that hinders their large-scale application, such as their high cost, low availability, and poor durability [5]. Hence, an extensive research has been carried out to develop new precious metal free catalysts capable to increase the use of fuel cells in industry.

One of the most promising alternatives to replace electrocatalysts based on noble metals are metal-free carbon materials doped with different heteroatoms (N, P, S, etc.) [6,7,8]. These materials have low cost, high surface area, and good mechanical and electrical properties. Moreover, they display high chemical and electrochemical stability [9,10]. In particular, N-doped carbon materials have demonstrated outstanding electrocatalytic properties [11]. The presence of nitrogen can modify the electron–donor properties of the carbon material and provides a redistribution of electronic density, producing an increase of the electrocatalytic activity for the ORR. However, the role of the different nitrogen functional groups is not still well understood [12]. Moreover, several works have pointed out the enhancement of electrocatalytic performance when increasing the surface area of the carbon material, either working as catalyst support or catalyst itself [13]. Thus, the synthesis of new carbon materials with large surface area and specific nitrogen functionalities appears as a promising approach for the development of new electrocatalysts for ORR.

Nitrogen-containing porous carbon materials can be synthesized by following different methods [14]. They are summarized as follows:

(i) Carbonization of a nitrogen-containing precursor (such as pyridine, melamine, and polyaniline), which can be followed by an activation process (either physical or chemical).
(ii) Hydrothermal carbonization of nitrogen containing-compounds (glucosamine, cyanuric acid, etc.).
(iii) Templating approaches using a nitrogen-containing precursor followed by a thermal treatment.
(iv) Post-thermal treatments of a material previously synthesized with a nitrogen-containing reactant in gas or liquid phase.

The first method has been extensively employed to produce N-doped carbon materials derived from polyaniline with large nitrogen content [15]. These carbon materials display outstanding electroactivity as catalyst supports [16,17,18] and good response when working themselves as electrocatalysts towards ORR [19,20,21,22,23,24,25]. The well-defined structure of polyaniline leads to the formation of carbon materials with predominance of certain types of functionalities depending on the treatment temperature [20]. Moreover, polyaniline has been extensively used to prepare hybrid materials by chemical or electrochemical polymerization of aniline over a carbon support [15]. These composites can be converted into advanced N-doped carbons by thermal post-treatments [15,26]. This strategy allows the production of carbon materials with a surface chemistry characteristic of polyaniline-derived carbon materials but which maintain most of the porous structure of the pristine carbon support.

Another promising methodology to generate N-doped carbon materials with high surface area is based on chemical post-treatments through organic chemistry reactions at low temperature [27,28]. In this procedure, nitrogen doping only depends on the reactivity between the carbon surface (i.e., oxygen functional groups) and the nitrogen-containing reaction medium. This approach leads to the anchoring of a wide range of nitrogen functionalities. Due to the mild conditions of the treatment (i.e., chemical functionalization at low temperature), nitrogen groups with low thermal stability (i.e., amides, amines) are generated on these carbon materials. Thus, the surface chemistry of these carbons can also be easily tuned by selective post-thermal treatments under inert atmosphere.

Herein, we report the performance of N-doped superporous activated carbons as electrocatalysts for the ORR. An activated carbon with very well developed microporosity (S_BET_ > 3000 m^2^/g) was used as starting material for further functionalization. The samples are obtained by different N-doping pathways: (i) chemical polymerization of aniline; (ii) nitrogen functionalization by organic reactions; and (iii) thermal post-treatments of the samples obtained with the pathways (i) and (ii). The combination of these strategies allows us to preserve most part of the porous texture of the pristine carbon material while the surface chemistry is severely modified [26,29]. The methodology used in this study leads to the synthesis of carbon materials with very large apparent surface area and similar porous texture, but their surface chemistry is systematically modified using a step-by-step protocol. This is an important difference with respect to a previous study [20], in which the materials were prepared by direct carbonization of polyaniline under inert atmosphere, resulting in materials with much lower surface area. Thus, these activated carbons can be used to get information about the role of different nitrogen functionalities in the electroactivity for the ORR for materials with a high development of porosity.

## 2. Materials and Methods

### 2.1. Synthesis of Activated Carbons

#### 2.1.1. Pristine Activated Carbon

A superporous activated carbon synthesized in our laboratory has been used as the starting material for nitrogen functionalization by different methods. The pristine material (named as KUA), has been obtained by chemical activation of a Spanish anthracite with KOH using a ratio of activating agent to raw material of 4:1, a nitrogen flow of 800 mL/min and an activation temperature of 750 °C under inert atmosphere, which was held for 1 h. More details about the preparation process are available elsewhere [30].

#### 2.1.2. Chemical Functionalization of Activated Carbon at Mild Conditions

KUA was further functionalized with nitrogen functional groups by two different strategies based on the organic chemistry protocols. Briefly, the first approach consisted in a three-step protocol: (i) chemical oxidation with HNO_3_, (ii) treatment with SOCl_2_, and (iii) amidation reaction with NH_4_NO_3_/DMF and pyridine. The obtained sample was named as KUA-CONH_2_. In the second method, the third step of the first protocol is directly applied over pristine sample (KUA-N). This sample was named as KUA-N. This methodology was accurately described in our previous works [27,28].

#### 2.1.3. Preparation of Polyaniline/Activated Carbon Composite

PANI/KUA composite was prepared by chemical polymerization of aniline as described elsewhere [31]. The aniline (solution of 70 mM) was adsorbed on the carbon material during 24 h at 30 °C. Afterwards, the sample was treated with ammonium persulfate solution in 1 M HCl for 1 h in a reactor at 0 °C. The aniline monomer:ammonium persulfate molar ratio was 1:1. The composite was washed with 1M HCl, 1M NH_4_OH and distilled water, and finally dried under vacuum for 24 h.

#### 2.1.4. Post-Thermal Treatments

**Scheme 1:**
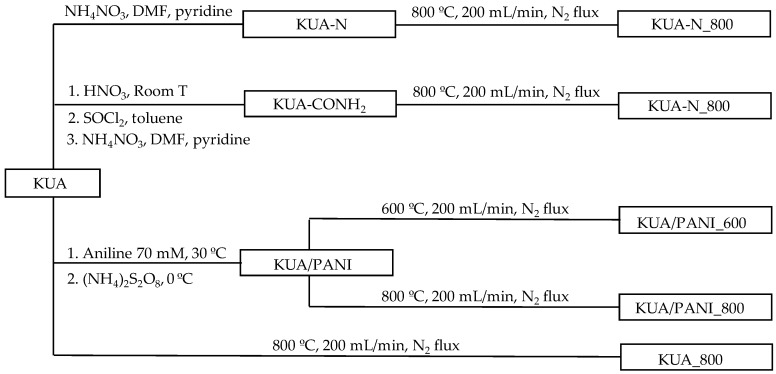
Summary of synthesis conditions used for the preparation of the carbon materials.

The carbon materials (KUA, KUA-CONH_2_ and KUA-N samples) and PANI/KUA composite were heat-treated at different temperatures under N_2_ atmosphere using (200 mL/min, 1 h) and a heating rate of 5 °C/min. The obtained samples are named as S_T, where S is the pristine sample (KUA, KUA-N, KUA-CONH_2_ and KUA/PANI) and T is the final heating temperature (600 and 800 °C). The summary of the synthesis methods and carbon materials prepared in this work are summarized in Scheme 1.

### 2.2. Porous Texture and Surface Chemistry Characterization

The porous texture characterization was carried out by N_2_ adsorption-desorption isotherms at −196 °C and by CO_2_ adsorption at 0 °C by using an Autosorb-6-Quantachrome apparatus (Quantachrome Instruments, Boynton Beach, FL, USA). The samples were outgassed at 200 °C for 4 h before the experiments. The apparent surface area was obtained from N_2_ adsorption isotherms using the BET equation in the 0.05-0.20 range of relative pressures. The total micropore volume was determined by Dubinin–Radushkevich (DR) method applied to N_2_ (relative pressures from 0.01 to 0.05) adsorption isotherms. The volume of the narrow microporosity (i.e., pore sizes below 0.7 nm) was calculated from the DR method applied to the CO_2_ adsorption isotherms (relative pressures from 0.0001 to 0.25) [32]. The pore size distribution of the samples was calculated from the N_2_ adsorption isotherms using the 2D-NLDFT Heterogeneous surface model [33] and by applying the Solution of Adsorption Integral Equation Using Splines (SAIEUS, available online at http://www.nldft.com/) Software.

The surface chemistry of the samples was analyzed by X-ray Photoelectron Spectroscopy (XPS) and Temperature Programmed Desorption (TPD). XPS measurements were performed by using a VG-Microtech Multilab 3000 spectrometer (Thermo-Scientific, Waltham, MA, USA), equipped with an Al anode. The deconvolution of N1s spectrum was carried out by using Gaussian functions with 20% of Lorentzian component. FWHM of the peaks was kept between 1.4 and 1.7 eV and a Shirley line was used for estimating the background signal. TPD experiments were performed by heating the samples (~10 mg) to 950 °C (at a heating rate of 20 °C/min) under a helium flow rate of 100 mL/min. The analyses were carried out using a TGA-DSC instrument (TA Instruments, SDT Q600 Simultaneous, New Castle, DE, USA) coupled to a mass spectrometer (Thermostar, Balzers, GSD 300 T3, Asslar, Germany).

### 2.3. Electrochemical Activity towards ORR

The evaluation of electroactivity towards ORR was carried out in a three-electrode cell using an Autolab PGSTAT302 bipotentiostat (Metrohm Autolab, Utrecht, The Netherlands). A rotating ring-disk electrode (RRDE, Pine Research Instrumentation, Burham, NC, USA) equipped with a glassy carbon disk (5.61 mm diameter) and a platinum ring was used as the working electrode. A platinum wire was used as the counter electrode and a reversible hydrogen electrode (RHE) as reference electrode. Additional experiments were done using graphite as counter electrode to discard any effect from using Pt counter electrode. The catalysts were dispersed by ultrasonication in ethanol-water mixture and drop-casted over the glassy carbon disk. The dispersion was prepared by mixing the carbon material with ethanol and Nafion (1.5% Nafion® perfluorinated resin solution (in water), Sigma Aldrich), in order to obtain a concentration of 2 mg/mL (of carbon material in ethanol and water). The amount of catalyst was optimized to reach the highest limiting current. The optimum value was determined to be 240 µg. The catalysts were deposited by dropping 120 µL of a 2 mg/mL dispersion made of each sample, obtaining a catalyst charge of 0.96 mg/cm^2^.

The electrochemical tests were carried out by cyclic voltammetry (CV) and linear sweep voltammetry (LSV) in the potential range 0.0–1.0 V versus RHE, starting from the most positive potential (i.e., 1.0 V) to less positive potentials as corresponds to a reduction process. CVs were performed in a N_2_-saturated and O_2_-saturated atmosphere at 5 mV/s. LSV experiments were conducted in an O_2_-saturated atmosphere at different rotation rates (between 400 and 2025 rpm) at 5 mV/s. The temperature was kept at 25 °C during the experiment. The potential of the Pt ring electrode was held at 1.5 V versus RHE during LSV experiments. The electron transfer number (n) and the produced H_2_O_2_ (%) were determined from the RRDE measurements:

(1) $$n=\;\frac{4\;I_{d}}{I_{d}+\;I_{r}/N\;}$$

(2) $$\%\;H_{2}O_{2}=100\times \frac{2I_{R}}{I_{D}N\;+\;I_{R}}=100\times \frac{4-n}{2}$$

where *I_r_* and *I_d_* are the currents measured at the ring and the disk, respectively, and *N* is the collection efficiency of the ring, which was experimentally determined to be 0.37 [34]. The onset potential of the reaction is determined as the potential at which the current measured is −0.05 mA·cm^−2^.

## 3. Results and Discussion

### 3.1. Surface Chemistry Characterization

The effect of the different nitrogen functionalization treatments was analyzed by XPS and TPD. Table 1 summarizes the surface composition of the carbon materials. The effect of nitrogen doping in the synthesis of KUA-N and KUA-CONH_2_ at mild conditions was discussed in our previous study [27,28]. For comparison purposes, the data related to these samples and their derived heat-treated samples (KUA-CONH_2__800 and KUA-N_800) are included in Table 1. Briefly, the post-functionalization treatment produces the decrease of oxygen functionalities of the pristine carbon material because of the attachment of different nitrogen moieties. Thus, KUA-N sample has lower oxygen content than the pristine carbon material and it contains nitrogen functionalities derived from the reaction with CO-evolving groups, such as amines and nitrogen heterocycles [28]. The synthesis of KUA-CONH_2_ involves a pre-oxidation treatment to increase the amount of oxygen functional groups (CO_2_ and CO-evolving groups), which afterwards react to produce nitrogen functionalities. Consequently, this sample has contribution of nitrogen groups derived from CO_2_ evolving groups (amide-like functionalities) and from CO-evolving groups (amines, imines, nitrogen heterocycles) [27]. Moreover, KUA-CONH_2_ has higher oxygen content than the pristine carbon material because the oxidized pristine sample was the one used to apply the functionalization method.

**Figure 1:**
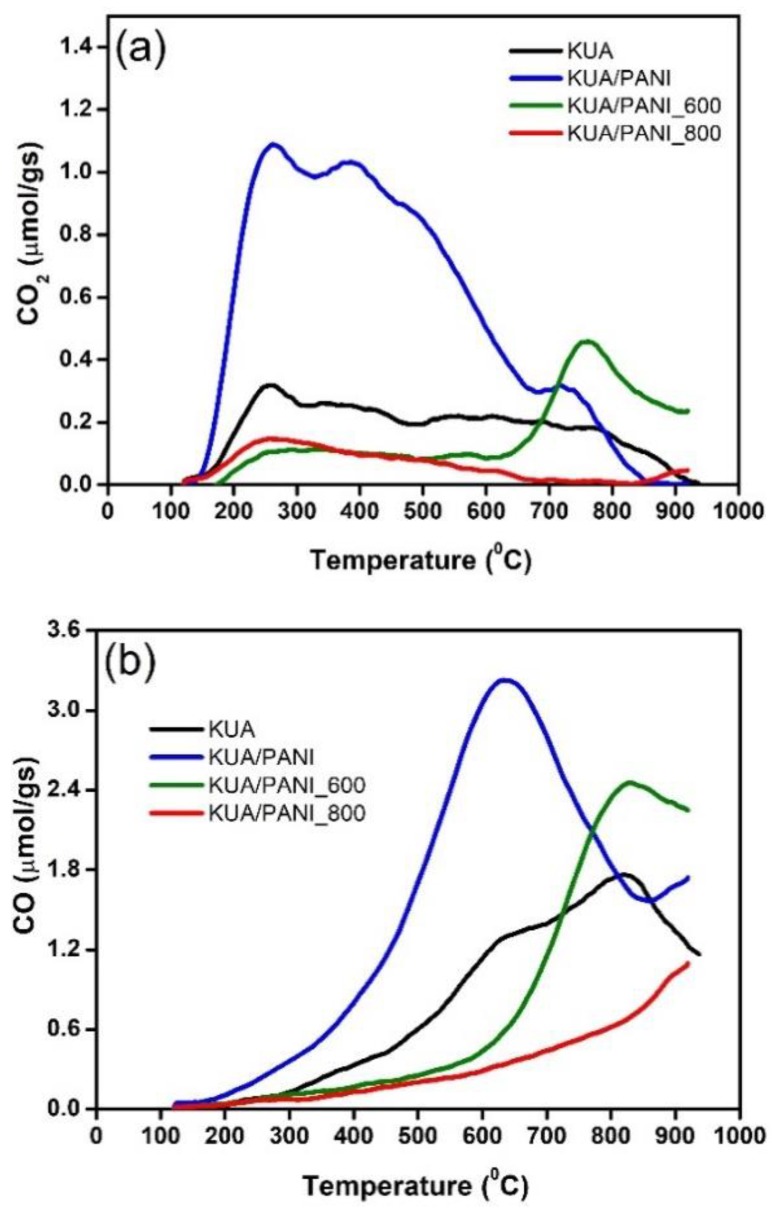
Comparison between (**a**) CO_2_ and (**b**) CO TPD profiles of KUA, KUA/PANI, KUA/PANI_600 and KUA/PANI_800.

The polymerization of aniline over KUA produces the generation of a polyaniline film inside the microporosity of the carbon material [31,35]. The attachment of nitrogen was confirmed by XPS (6.0 at.% N, Table 1). KUA/PANI composite also evidences an increase of oxygen content, as confirmed by XPS and TPD. Figure 1 illustrates the TPD profiles obtained for KUA, KUA/PANI composite and the derived heat-treated samples (KUA/PANI_600 and KUA/PANI_800). KUA/PANI composite displays larger amount of CO_2_ (carboxylic acids, lactones and anhydrides) and CO evolving groups (phenols, quinones, etc.) [36]. This is related to the use of an oxidizing agent on the polymerization of aniline in presence of the carbon material that may react with the carbon surface to produce oxygen functional groups.

The effect of heat treatments over the pristine and N-doped functionalized activated carbons, was previously studied and it was observed that the heat treatment diminishes most of the oxygen and nitrogen functional groups in KUA-CONH_2_ and KUA-N materials [29]. Similarly, the treatments over the composite strongly decrease the amount of oxygen functional groups. At 600 °C, ~71% of CO_2_ evolving groups are removed from KUA/PANI composite and the amount of CO evolving groups is severely decreased. These modifications lead to the generation of a N-doped activated carbon with similar oxygen content than the pristine sample (2620 and 2870 µmol/g for KUA/PANI_600 and KUA, respectively, Table 1). Nevertheless, the type of functionalities is different, being remarkably lower the concentration of groups that thermally desorb at temperatures lower than around 600 °C (i.e., phenols) [36]. It is also worth noting that the concentration of carbonyl type groups (above 600 °C) is larger in KUA/PANI_600 than in the composite, indicating that the heat treatment produces the conversion of phenols into carbonyl groups upon heating at 800 °C.

The largest oxygen content among the N-containing samples treated at 800 °C, is detected for KUA/PANI_800 (1050 µmol/g, Table 1). This can be explained by the presence of higher concentration of moieties in the parent composite (KUA/PANI sample), that may lead to higher reactivity between the carbon surface and the PANI coating upon heating.

**Figure 2:**
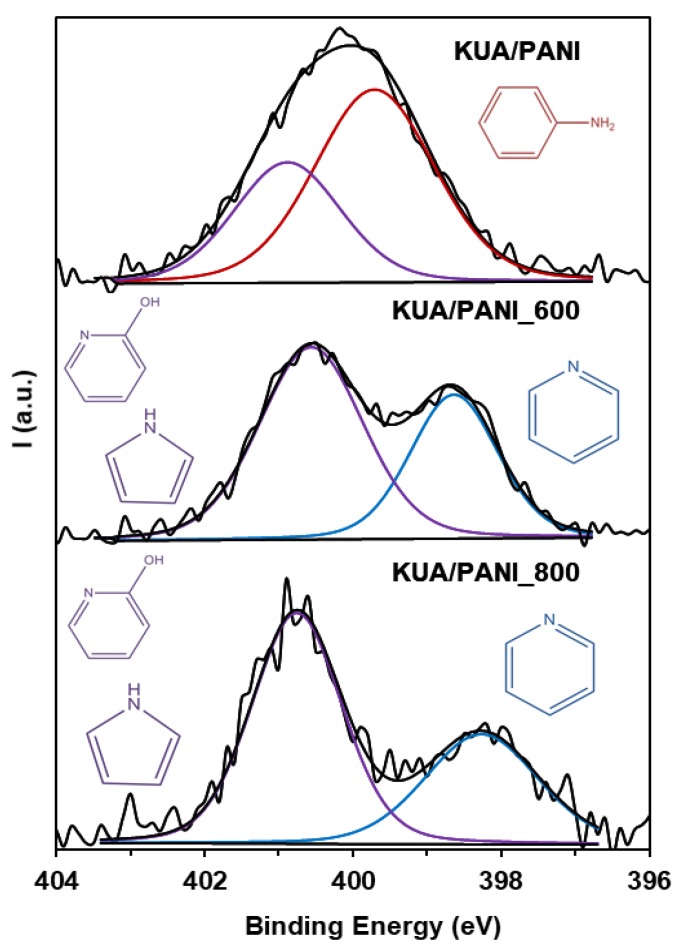
N1s XPS spectra deconvoluted for the KUA/PANI composite and the N-doped activated carbons.

The nitrogen functionalities are also modified as consequence of the heat treatments. Figure 2 shows the deconvoluted N1s XPS spectra obtained for KUA/PANI composite and the related heat-treated samples. The assignment of the different nitrogen functionalities is collected in Table 2. The values obtained for the other N-doped samples are included for comparison purposes. KUA/PANI composite exhibits a main peak associated to the presence of amines at 399.7 ± 0.2 eV [37,38,39]. Moreover, there is also a contribution at higher binding energy that can be assigned to positively charged N atoms (400.8 ± 0.2 eV) that can be due to reactions with oxygen functionalities during the polymerization of aniline [31]. The carbonization at 600 °C produces the decrease of nitrogen groups as well as their conversion into different surface functionalities. Thus, KUA/PANI after treatment at 600 °C shows two different peaks related to pyrroles/pyridones (400.6 ± 0.2 eV) and pyridines/imines (398.6 ± 0.2 eV) [37,38,39]. After heat treatment at 800 °C, the main contribution of N-functional groups is detected at 400.8 ± 0.2 eV, related to pyrroles/pyridones (70% N, Table 2), and a lower presence of pyridines/imines (398.3 ± 0.2 eV, 30% N, Table 2). These results are in agreement with the mechanism proposed for the carbonization of PANI [40,41]. During the heat treatment the polymer chain experiences bond breaking that, upon heating at higher temperatures, react again to form N-containing heterocycles (pyrroles/pyridones and pyridines) through cross-linking reactions. Thus, the amines present on KUA/PANI composite may experience degradation upon heating and react to generate nitrogen groups with higher thermal stability, such as nitrogen heterocycles.

KUA-N and KUA-CONH_2_ also experience loss and conversion of their nitrogen functionalities into groups with higher thermal stability [42]. At 800 °C, there are two main contributions of pyridine (398.7 ± 0.2 eV, Table 2) and pyrrole/pyridone (400.7 ± 0.2 eV, Table 2). However, the concentration of pyrrole/pyridone groups is lower than that found for KUA/PANI_800. Hence, the use of different surface modification methods and post-thermal treatments allows to obtain activated carbons with very different surface chemistry.

### 3.2. Porous Texture Characterization

**Figure 3:**
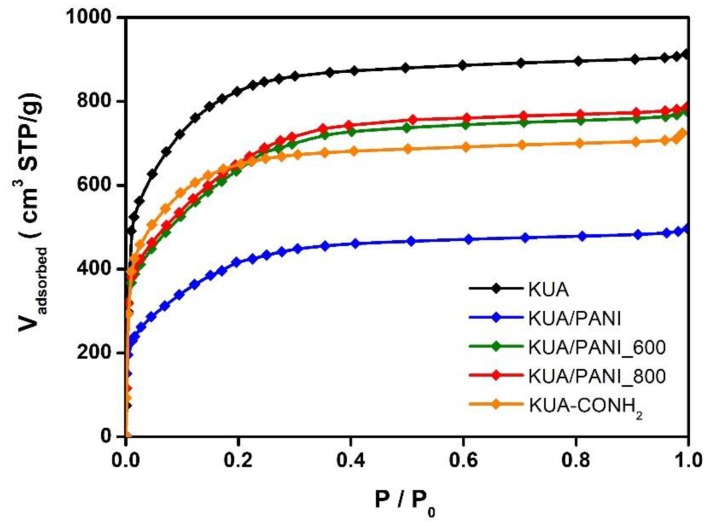
N_2_ adsorption isotherms obtained for KUA (pristine sample), KUA/PANI composite and N-doped activated carbons.

The porous texture of the samples was analyzed by N_2_ and CO_2_ adsorption isotherms. Figure 3 illustrates the N_2_ adsorption isotherms obtained for the pristine activated carbon and the PANI-derived carbon materials. For comparison purposes, the isotherm of KUA-CONH_2_ was also included. All samples evidence a type I isotherm characteristic of microporous materials. The pore size distribution of the carbon materials was also analyzed and is included in Figure S1.

Table 3 compiles data of the porous texture of the samples. The effects of nitrogen doping at mild conditions were thoroughly discussed in our previous works [27,28]. The functionalization at mild conditions produces some minor modifications on the microporosity of the samples. When using a single step modification protocol (for the production of KUA-N), the microporosity of the pristine carbon materials is fully retained [28]. However, the combination of oxidation process and amidation reactions leads to some loss of the apparent surface area, due to the generation of surface functionalities that occupies or block some part of the microporosity [27].

Regarding KUA/PANI composite, the formation of polyaniline layer over the pristine carbon material KUA increases the amount of nitrogen and oxygen functionalities (above mentioned) but produces the most important decrease of the apparent surface area and micropore volume (Table 3). After the heat treatments, the obtained activated carbons (KUA/PANI_600 and KUA/PANI_800) recover part of the microporosity, due to the decomposition of PANI layer. Hence, all heat-treated samples display similar apparent surface area (2400–2800 m^2^/g, Table 3). However, PANI-derived samples have around ~25% lower micropore volume than N-doped heat-treated carbons (KUA-N and KUA-CONH_2_). This is a consequence of the carbon layer formed in the heat-treated substrate from PANI decomposition. Thus, these modifications treatments allow to modify the surface chemistry while keeping most of the microporosity of the pristine carbon material.

### 3.3. Electroactivity Towards ORR

**Figure 4:**
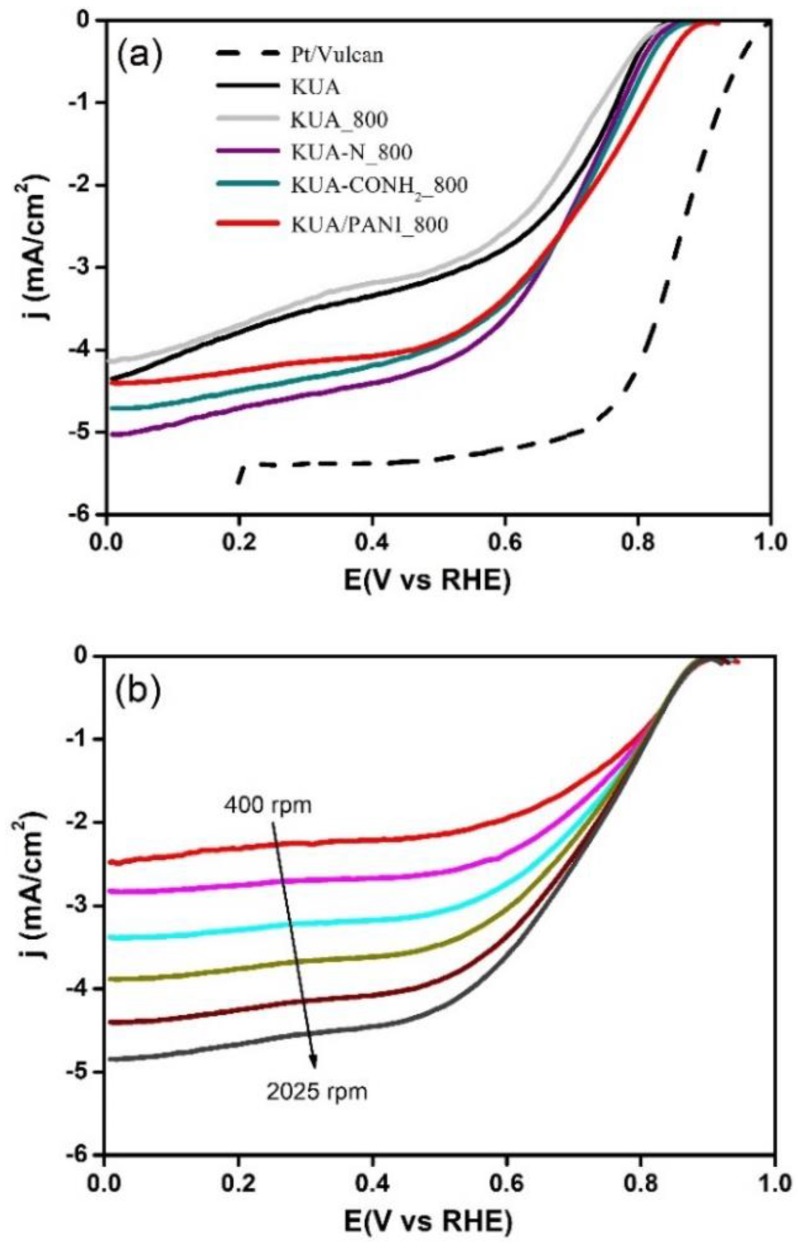
(**a**) Linear sweep voltammetry (LSV) curves for the catalysts in O_2_-saturated 0.1 M KOH at 1600 rpm. (**b**) LSV curves at different rotating rates for KUA/PANI_800. v = 5 mV/s.

The electroactivity of the carbons towards the ORR was analyzed in alkaline electrolyte (0.1 M KOH) by using a RRDE. LSV curves were measured at different rotating rates for all samples. The contribution of electrical double layer was subtracted by recording CV in N_2_-saturated 0.1 M KOH. Figure 4 shows the LSV curves obtained for some samples at 1600 rpm. The LSV for commercial 20% Pt/Vulcan was included for comparison purposes. Table 4 collects the main properties related to the performance of the samples as electrocatalysts for the ORR derived from the LSV curves.

#### 3.3.1. Pristine Activated Carbon

The pristine carbon material evidences a two-wave curve characteristic of microporous carbon materials [13]. The high onset potential (0.81 V, Table 4) demonstrated by this electrocatalyst can be explained by the high concentration of edge sites provided by its well-developed microporosity [13], which have been proposed as electrocatalytic sites [43,44,45,46,47,48].

#### 3.3.2. N-Doped Activated Carbons at Mild Conditions

The effect of nitrogen functionalities was thoroughly analyzed. The catalysts obtained by chemical methods (KUA-N and KUA-CONH_2_) have not improved electrocatalytic properties in comparison with the pristine carbon material (KUA). The onset potential was slightly decreased in both samples. Moreover, the limiting current was severely diminished for KUA-CONH_2_ sample (Figure S2). The poor performance of this material (KUA-CONH_2_) cannot only be related to the modification of its surface chemistry. In fact, this carbon material has N-heterocycles (pyridines, pyrroles/pyridones), which have been proposed as electrocatalytic sites towards ORR [8,19,23,24,25,49,50]. Thus, an improvement of the electrocatalytic response would be expected on this sample considering the existence of electrocatalytic functional groups. Moreover, the decrease on the performance cannot be associated with a decrease of conductivity since we have previously shown that it is enhanced in different electrolytes [27,29]. Hence, the poor electrocatalytic behavior might be a consequence of the surface modification protocol used to functionalize these samples. It is worth noting that the post-modification methods employed in this work might affect exclusively the edge sites of the graphene layer (i.e., the basal plane might not be modified), since the attachment of nitrogen moieties is only consequence of the reactivity between the functional groups at the carbon edge sites and the nitrogen-containing medium. Thus, the modification of the electroactivity of the electrocatalysts seems to be related to changes in the carbon edge sites. Regarding KUA-CONH_2_ carbon, the oxidation process involves the generation of oxygen functionalities (which are attached to the carbon edge sites). Thus, if the functionalization method does not produce exclusively nitrogen groups with higher electroactivity (i.e., pyridones, edge type N-Q), the N-doped sample might result as a worse electrocatalyst, since a noticeable amount of edge sites is lost during the surface modification reaction. Hence, the sample experiences a deactivation during the functionalization treatment.

A similar effect is expected for KUA-N sample, even though the oxidation process is avoided. It is expected that nitrogen functional groups are anchored to the carbon surface by consumption of oxygen functionalities. However, the mechanism might affect or involve the reactivity of the adjacent carbons. This effect was also proposed by Tuci et al. [51]. The electrografting of pyridine functional groups to N-doped carbon nanotubes resulted in a decrease of inherent electrochemical properties and “switched them off” towards ORR. Thus, functionalization at mild conditions might produce also a deactivation of the carbons as electrocatalysts.

#### 3.3.3. Polyaniline/Activated Carbon Composite

The electroactivity towards ORR of KUA/PANI composite was also evaluated. Table 4 compiles the onset potential recorded for this sample. This material displays lower onset potential and limiting current than pristine activated carbon (see Figure S2). This poor performance is explained by its surface chemistry (see Table 1 and Table 2). This composite has a large concentration of amine functionalities, which are not electroactive towards ORR [50]. Moreover, the formation of the polyaniline film over the microporosity of the carbon surface decreases the apparent surface area (see Section 2.2). Although the microporosity is still very large (0.54 cm^3^/g) in KUA/PANI composite, this value is ~50% of that of the pristine carbon material (KUA). Hence, the loss of microporosity might also diminish the concentration of accessible edge sites, resulting in an overall decrease of the catalytic electroactivity of the composite.

#### 3.3.4. Heat-Treated Activated Carbons

The electrocatalytic performance of the carbon materials is improved for the N-doped samples after heat treatment at 800 °C (KUA-N_800, KUA-CONH_2__800 and KUA/PANI_800). All the electrocatalysts display higher onset potential, being the largest value for KUA/PANI_800 (0.88 V versus RHE). Moreover, these carbons provide higher limiting currents than the other samples, including the pristine KUA sample and the heat treated KUA_800 (Figure 4a).

Since the parent non-doped carbon (KUA_800) does not show any enhancement of the catalytic response (Figure 4a), the increase of the performance observed for the N-containing carbons after treatment at 800 °C is not only related to the effect of heat treatment. This enhancement of the electrocatalytic response for the ORR is undoubtedly related to the existence of nitrogen functional groups. The N-doped carbons have similar content of pyridines (0.5–0.6 at.% XPS, Table 1) and pyrroles/pyridones (0.9–1.4 at.% XPS, Table 1). The main difference on the nitrogen functional groups arises for KUA/PANI_800, whose content of pyrroles/pyridones is larger (1.4 at.% XPS). Moreover, this material has higher content of CO-evolving groups (Table 1), being more likely the presence of pyridones. The improvement of the ORR through pyridones (N–C–O sites) was already proposed in the literature [8,19,21].

The enhancement of ORR by pyridine-containing carbon catalysts has been also proposed [8,23,24,25,49], but there is controversy about its positive effect [52]. Nevertheless, the three samples display similar pyridine content, and consequently this functional group cannot be responsible of the large improvement observed in KUA/PANI_800. Its enhanced electrocatalytic properties might be related to the pyridone functional groups.

It is also worth noting that in spite of the fact that KUA/PANI_600 has higher amount of N-species and similar porous texture in comparison with KUA/PANI_800, its performance towards ORR is significantly poorer (Figure S2). This is related to the lower conductivity of KUA/PANI_600 compared to KUA/PANI_800) [20].

#### 3.3.5. Selectivity to Water Formation

**Figure 5:**
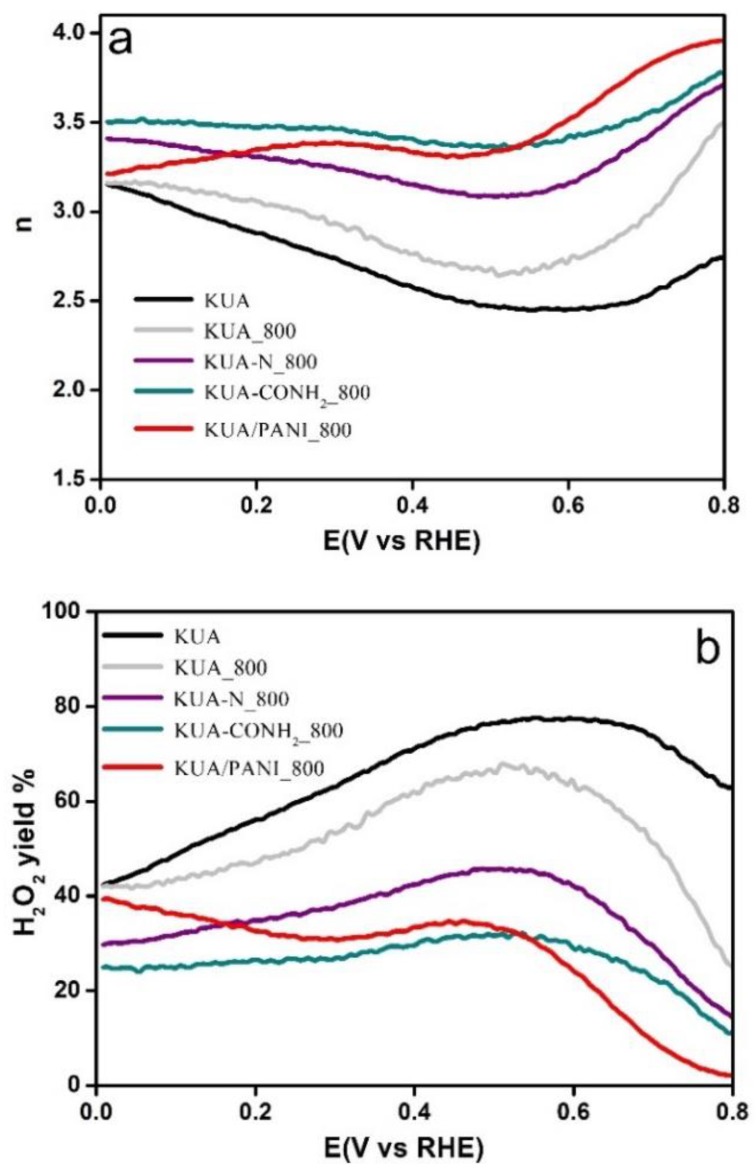
(**a**) Electron transfer number and (**b**) H_2_O_2_ yield calculated from RRDE experiments of activated carbon electrocatalysts in O_2_-saturated 0.1 M KOH at 5 mV/s and 1600 rpm.

The electron transfer number involved in the ORR was determined by monitoring the current registered in the Pt ring electrode during the experiments. Table 4 collects the values at 0.5 V registered for all samples. Figure 5a illustrates the evolution of the number of electrons with the potential for a selection of samples and Figure 5b shows the evolution of H_2_O_2_ during the experiment. The pristine carbon material (KUA) catalyzes the ORR through a mechanism in two steps [13]. The first one occurs at high potentials and involves the reduction of oxygen to hydrogen peroxide. Afterwards, the hydrogen peroxide that remains in the microporosity of the activated carbon is subsequently reduced to hydroxide ions. Both steps happen through a 2e^−^ process. Hence, the increase of number of electrons observed for this sample when shifting the potential to more negative values is explained by the sum of the electrons involved in both reaction steps (2 + 2 e^−^ pathway) [13]. A recent study [53] claimed that the selectivity to water formation is enhanced in the sites of the basal plane, while the edge sites are selective to production of H_2_O_2_. However, since the large microporosity content of the activated carbon might produce a large concentration of both edge and planar sites, the low number of electrons cannot be explained attending to this explanation.

The N-doped carbons heat-treated at 800 °C involve the largest number of electrons for the ORR among all samples (see Table 4). Specifically, the number measured at 0.5 V is 3.4, 3.4, and 3.1 for KUA/PANI_800, KUA-CONH_2__800, and KUA-N_800, respectively. This points out a higher selectivity towards the formation of water in comparison with the non-doped carbons. For comparison purposes, the measurement of KUA_800 (non-doped and heat-treated pristine sample) was also included in Figure 5. This sample shows an enhancement of the number of electrons along the whole range of potentials in comparison with the pristine sample (KUA). However, its selectivity to water formation is lower than that demonstrated by the N-doped ones heat-treated at 800 °C. Hence, the enhancement manifested by these materials is related to the formation of N-functional groups with higher electrocatalytic activity for the ORR. These samples have large content of pyridones (N–C–O sites), which have been proposed to enhance the selectivity to water formation [19,21]. Moreover, the N-doped carbons do not show the important increase of number of electrons (to 2 +2e^−^ pathway) displayed by the non-doped samples at low potentials. The result is especially interesting in case of KUA/PANI_800, since the number of transferred electrons decreases as the potential decreases and the values at the highest potentials are closer to 4 (i.e., at around 0.7 V). Thus, this carbon material shows the highest selectivity to water formation compared to the other N-doped samples.

## 4. Conclusions

In this study, several N-doped superporous activated carbons (1–4 at.% XPS) were prepared by following different post-functionalization treatments, including methods involving organic chemistry pathways, polymerization of aniline and post-thermal treatments. The combination of these strategies leads to the production of activated carbons with large apparent surface area and different surface chemistry. The obtained samples have different functional groups depending on the post-modification treatment, such as moieties with low thermal stability (amines, amides) and groups with high thermal stability (pyrroles, pyridines, etc.). Thus, they have been used to monitor the effect of nitrogen functional groups on the performance of highly microporous activated carbons as electrocatalysts for ORR.

The pristine sample displays remarkable electroactivity towards ORR due to its well-developed microporosity. The N-doped activated carbons evidence different electrocatalytic performance depending on the functionalization strategy. The doping methods at low temperature produce a decrease of the catalytic response due to the generation of non-catalytic active sites (amines, amides) and the decrease of catalytic edge sites. The performance is improved for N-doped activated carbons after heat treatment at 800 °C as consequence of the generation of electrocatalytic nitrogen-containing functional groups. Polyaniline-derived activated carbon (heat-treated at 800 °C) provided the highest electroactivity (onset potential of 0.88 V) and improved selectivity to water formation. This enhanced behavior is explained by its highest concentration of N–C–O sites.

## Figures and Tables

**Table 1:** Surface composition of the activated carbons obtained by Temperature Programmed Desorption (TPD) and X-ray Photoelectron Spectroscopy (XPS).

Sample	CO_2_ (µmol/g)	CO (µmol/g)	O (µmol/g)	O_XPS_ (at.%)	N_XPS_ (at.%)
KUA	450	1970	2870	8.8	0.3
KUA_800	170	620	960	2.3	-
KUA/PANI	1320	3560	6200	11.4	6.0
KUA/PANI_600	380	1860	2620	4.5	4.7
KUA/PANI_800	210	740	1050	6.7	2.0
KUA-CONH_2_	1140	2370	4650	10.2	4.2
KUA-CONH_2__800	140	570	830	4.5	2.1
KUA-N	450	1750	2640	7.5	3.7
KUA-N_800	130	505	720	3.0	1.7

**Table 2:** Assignment of N1s deconvoluted curves to nitrogen functional groups.

Sample	Binding Energy (eV)	Functional Group	N (at.%)	Percentage of N Species
KUA/PANI	400.8 ± 0.2	Positive N	2.2	36
	399.7 ± 0.2	Amines	3.8	64
KUA/PANI_600	400.6 ± 0.2	Pyrrole, pyridone	2.9	61
	398.6 ± 0.2	Pyridine, Imine	1.8	39
KUA/PANI_800	400.8 ± 0.2	Pyrrole, pyridone	1.4	70
	398.3 ± 0.2	Pyridine, imine	0.5	30
KUA-N	401.9 ± 0.2	Quaternary	0.4	10
	400.7 ± 0.2	Pyrrole,pyridone	0.9	25
	399.8 ± 0.2	Amide, Lactam, Amine, Imide	1.3	35
	398.7 ± 0.2	Pyridine, Imine	1.1	30
KUA-N_800	402.7 ± 0.2	Oxidized N	0.2	14
	400.8 ± 0.2	Pyrrole, pyridone	0.9	51
	398.7 ± 0.2	Pyridine, imine	0.6	35
KUA-CONH_2_	400.7 ± 0.2	Pyrrole, pyridone	0.7	19
	399.8 ± 0.2	Amide, lactam, amine, imide	1.9	50
	398.8 ± 0.2	Pyridine, imine	1.2	31
KUA-CONH_2__800	402.5 ± 0.2	Oxidized N	0.2	11
	400.8 ± 0.2	Pyrrole, pyridone	0.9	52
	398.7 ± 0.2	Pyridine, imine	0.6	37

**Table 3:** Porous texture of the samples.

Sample	S_BET_ (m^2^/g)	V_DR_N_2_ (cm^3^/g)	V_DR_CO_2_ (cm^3^/g)
KUA	3080	1.19	0.57
KUA_800	2680	1.05	0.49
KUA/PANI	1590	0.54	0.37
KUA/PANI_600	2420	0.81	0.49
KUA/PANI_800	2470	0.84	0.56
KUA-COOH	2770	1.06	0.49
KUA-CONH_2_	2390	0.97	0.45
KUA-CONH_2__500	2630	1.02	0.41
KUA-CONH_2__800	2630	1.0	0.43
KUA-N	2960	1.18	0.52
KUA-N_500	2800	1.11	0.49
KUA-N_800	2770	1.09	0.48

**Table 4:** Electrochemical parameters of the electrocatalysts calculated from the rotating ring-disk electrode (RRDE) experiments in O_2_-saturated 0.1M KOH at 5 mV/s and 1600 rpm.

Sample	E_onset_ (V versus RHE)	n (at 0.5 V)
KUA	0.82	2.5
KUA_800	0.82	2.7
KUA/PANI	0.80	2.4
KUA/PANI_600	0.82	2.7
KUA/PANI_800	0.88	3.4
KUA-CONH_2_	0.79	2.6
KUA-CONH_2__800	0.85	3.4
KUA-N	0.81	2.8
KUA-N_800	0.84	3.1
Pt/Vulcan	0.98	3.9

## Supplementary Materials

The following are available online at https://www.mdpi.com/1996-1944/12/8/1346/s1, Figure S1: N_2_ adsorption 2D-NLDFT-PSD of all KUA-based samples, Figure S2: LSV curves for the catalysts in O2-saturated 0.1 M KOH at 1600 rpm. v = 5 mV/s.
