# Supplementary material for: Nitrogen-Doped Superporous Activated Carbons as Electrocatalysts for the Oxygen Reduction Reaction
Source: Materials (Basel). 2019 Apr 25;12(8):1346. doi: 10.3390/ma12081346 (PMC6515461; doi:10.3390/ma12081346)
Supplement: Supplementary file 1 [file materials-12-01346-s001.pdf]

materials

Supplementary Information

Nitrogen-Doped Superporous Activated Carbons as Electrocatalysts for the Oxygen Reduction Reaction

María José Mostazo-López [1], David Salinas-Torres [2], Ramiro Ruiz-Rosas [1], Emilia Morallón [2] and Diego Cazorla-Amorós [1,*]

[1] Department of Inorganic Chemistry and Materials Institute, University of Alicante, Alicante 03080, Spain; mj.mostazo@ua.es (M.J.M.-L.); ramiro@uma.es (R.R.-R.)

[2] Department of Physical Chemistry and Materials Institute, University of Alicante, Alicante 03080, Spain; david.salinas@ua.es (D.S.-T.); morallon@ua.es (E.M.)

* Correspondence: cazorla@ua.es; Tel.: +34-965-903-946

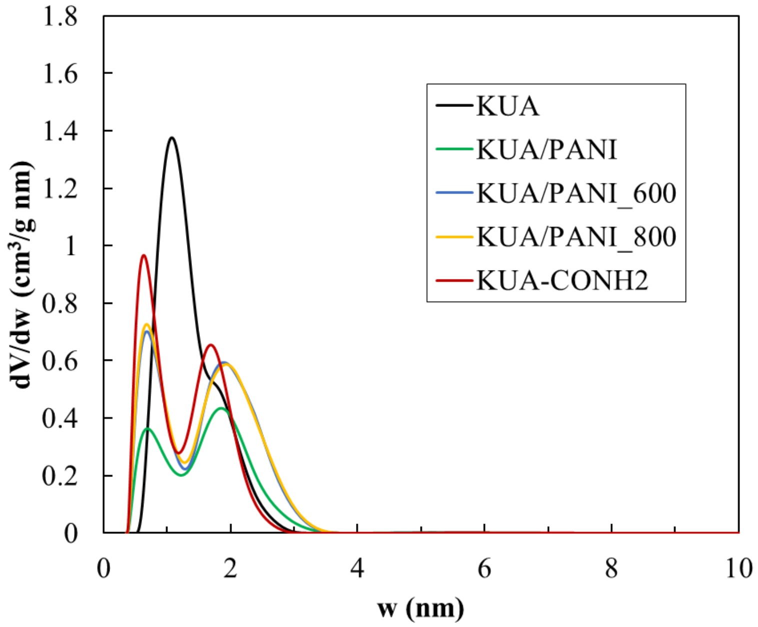

Figure S1. N_2 adsorption 2D-NLDFT-PSD of all KUA-based samples.

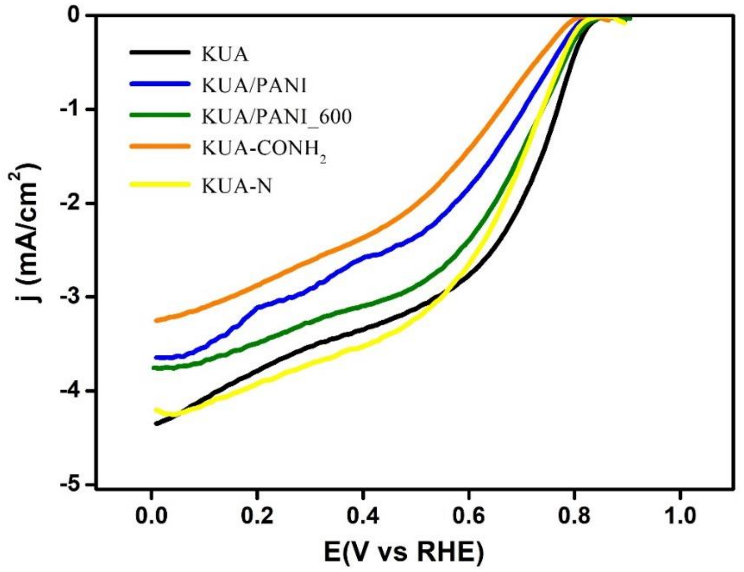

Figure S2. LSV curves for the catalysts in O2-saturated 0.1 M KOH at 1600 rpm. v = 5 mV/s.
